# Toward Green Production of Chewing Gum and Diet: Complete Hydrogenation of Xylose to Xylitol over Ruthenium Composite Catalysts under Mild Conditions

**DOI:** 10.34133/2019/5178573

**Published:** 2019-11-29

**Authors:** Cai-Juan Liu, Ning-Ning Zhu, Jian-Gong Ma, Peng Cheng

**Affiliations:** Department of Chemistry and Key Laboratory of Advanced Energy, Material Chemistry, College of Chemistry, Nankai University, Tianjin 300071, China

## Abstract

Xylitol is one of the most famous chemicals known to people as the essential ingredient of chewing gum and as the sugar alternative for diabetics. Catalytic hydrogenation of biomass-derived xylose with H_2_ to produce high-value xylitol has been carried out under harsh reaction conditions. Herein, we exhibit the combination of Ru NPs with an environmentally benign MOF (ZIF-67) to afford a heterogeneous composite catalyst. Complete conversion of xylose with 100% selectivity to xylitol was achieved at 50°C and 1 atm H_2_. This is the first successful attempt to produce xylitol with ambient pressure H_2_ as well as the first time to achieve a 100% selectivity of xylitol for applicable catalysts. We also proved the universality of the Ru@ZIF-67 towards other hydrogenation processes. Under 1 atm H_2_, we achieved 100% conversion and >99% selectivity of 1-phenylethanol at 50°C for the hydrogenation of acetophenone. This is also the first report of hydrogenating acetophenone to 1-phenylethanol under 1 atm H_2_, which confirms that our result not only contributes to enhance the industrial yields of xylitol and reduces both the economical and energy costs but also provides new perspectives on the other hydrogenation process with H_2_.

## 1. Introduction

Xylitol is one of the most famous chemicals known to people as the essential ingredient of chewing gum and as the sugar alternative for diabetics because this water-soluble sugar alcohol has higher sweetness yet lower calories and does not rely on insulin in comparison with sucrose. Nowadays, the conversion of renewable biomass into value-added chemicals and fuels has received increasing attention because of the depletion in fossil fuels and the deterioration due to global warming worldwide [1–5]. As a natural sweetener obtained by the hydrogenation of xylose [6–10], xylitol is recognized as one of the top twelve value-added chemicals from biomass by the US Development of Energy [1, 8–10]. Moreover, it is widely used in food, cosmetic, pharmaceutical, and synthetic resin industries [6, 7, 10–16]. The demand for xylitol is escalating owing to its various benefits and increasing health and weight consciousness of humans [6, 7, 14, 17]. The global consumption of xylitol is approximately 160000 metric tons with a market of US$ 670 million in 2013 and is expected to reach 242000 metric tons with US$ 1 billion by 2020s in a growth at a Compound Annual Growth Rate (CAGR) of 6.95% during the period 2017-2021 [14, 17].

Industrially, xylitol is produced by the hydrogenation of xylose, which is obtained from hydrolysis of xylan-containing plant relatives such as corncobs or hardwoods [1–7, 18]. Attempts to find suitable catalysts for the hydrogenation of xylose refer to supported noble metals (Pt, Pd, and Ru) or Ni on different supports such as zeolites, carbon, or alumina [1–16, 18, 19]. However, the aggregation of noble metal nanoparticles (NPs) or metal leaching from the supporter generates a negative impact on the catalytic activity and recyclability. Up to now, catalytic hydrogenation of xylose to xylitol can only be carried out under harsh reaction conditions (e.g., 100-300°C and up to 50 atm H_2_ pressure) [6, 7, 20], which is costly, energy-intensive, and environmentally unfriendly and causes high potential risk. Meanwhile, it is still a significant challenge to achieve high selectivity at raised conversion owing to the generation of numerous by-products [6, 7]. Consequently, new catalysts are in high demand for xylose hydrogenation with significantly improved catalytic activity and selectivity.

In recent years, increasing attention has been given to composite materials with metal NPs supported on metal-organic frameworks (MOFs) due to the combination of the activity of NPs with the large specific surface area, uniform pore sizes, and abundant structures of MOFs, which leads to significantly improved or even new performance in comparison with the pristine counterparts [21–24]. Herein, we exhibit the complete hydrogenation of xylose under unprecedentedly soft reaction conditions (1 atm H_2_ pressure and 50°C) through the combination of Ru NPs with an environmentally benign MOF (ZIF-67) as a new type of heterogeneous composite catalyst Ru@ZIF-67. We perform the first example of hydrogenating xylose to xylitol under 1 atm H_2_ pressure and obtain a 100% selectivity and excellent reusability of the composite catalyst, which is the first time to achieve a 100% selectivity of xylitol for applicable catalysts. This is also the first time to apply MOF composites for catalyzing the hydrogenation of xylose, as far as we know. Furthermore, as a bifunctional catalyst, Ru@ZIF-67 can enrich H_2_ molecules around Ru NPs to a certain level [25–28] and efficiently dissociate the H-H bond through highly dispersed and confined Ru NPs [6, 29–31], rendering it to be a universally valid catalyst for hydrogenation process, which is confirmed by achieving the hydrogenation of acetophenone to 1-phenylethanol under 1 atm H_2_ for the first time.

## 2. Results

### 2.1. Synthesis of ZIF-67

ZIF-67 was prepared according to the literature [32]. In a typical synthesis, 5.5 g of 2-methylimidazole was dissolved in 20 mL of deionized water. Then, 0.45 g Co(NO_3_)_2_·6H_2_O was dissolved into 3 mL of deionized water. Then, an aqueous solution of Co(NO_3_)_2_·6H_2_O was added dropwise to the 2-methylimidazole solution with stirring. After stirring for 6 h at room temperature, the solid was obtained from the solution by centrifugation and washed with water and methanol and dried at 353 K under vacuum.

### 2.2. Preparation of Ru@ZIF-67

In this experiment, a series of ZIF-67-supported Ru catalysts were prepared through a conventional liquid impregnation-reduction method described in the literature [23]. First, 200 mg of activated ZIF-67 powder and various amounts of ruthenium chloride (40 mg, 60 mg, and 80 mg) was dispersed into 4 mL of acetonitrile, and then the mixture was kept stirring for 24 hours at room temperature under an Ar atmosphere. After the impregnation, it was centrifuged and washed with CH_3_CN. The solid was dried at 100°C under vacuum. Then, it was reduced by the ethanol solution of sodium borohydride under Ar. The resultant was filtered, washed by ethanol, and finally dried at 100°C under vacuum. In this paper, Ru@ZIF-67 with different Ru loadings were denoted as *1a*, *1b*, and *1c*, respectively.

### 2.3. Catalytic Tests

The hydrogenation experiments were carried out in a Schlenk tube (10 ml) with one opening connected to a hydrogen balloon. In all experiments, a mixture of xylose (150 mg) and required amount of catalyst was charged into the reaction tube, which was sealed and pressurized with H_2_ at 1 atm. Subsequently, the solvent (5 mL) was added into the reaction mixture under stirring, and the reaction was carried out at 323 K under ambient atmosphere for 48 h. The reaction mixture was separated by filtration and analyzed by HPLC (Waters600E) equipped with a refractive index detector (25°C) and turner NH_2_ column. Acetonitrile water (85 : 15, volume ratio) was used as an eluent at a flow rate of 1 mL·min^−1^ at 25°C. The conversion of xylose and selectively to xylitol was calculated using the following methods [7, 9]:

(1) $$\begin{array}{c} \text{ Xylose conversion }\left(\%\right)=1-\frac{\text{Mole of xylose unconverted }\left(\text{HPLC}\right)}{\text{Initial mole of xylose}}\times 100, \end{array}$$

(2) $$\begin{array}{c} \text{Yield of xylitol }\left(\%\right)=\frac{\text{Mole of xylitol }\left(\text{HPLC}\right)}{\text{Initial mole of xylose}}\times 100, \end{array}$$

(3) $$\begin{array}{c} \text{Selectivity of xylitol }\left(\%\right)=\frac{\text{Mole of xylitol }\left(\text{HPLC}\right)}{\text{Mole of all products formed}}\times 100. \end{array}$$

### 2.4. Catalyst Characterization

The three-dimensional cobalt-based MOF ZIF-67 was selected as the matrix for uploading Ru NPs, due to its large surface area, easy synthesis, and especially higher H_2_ adsorption performance over the other series of MOFs [25–28, 33–40]. ZIF-67-supported Ru catalysts Ru@ZIF-67 with different Ru contents were synthesized via a simple impregnation-reduction strategy and were labeled as *1a*, *1b*, and *1c* according to the Ru contents of 9.30, 11.9, and 15.6 wt% revealed by inductively coupled plasma (ICP) analyses, respectively. Powder X-ray diffraction (PXRD) patterns of *1a*–*1c* showed good agreement with that of parent ZIF-67, indicating the integrity of the ZIF-67 framework during the uploading process ([Supplementary-material supplementary-material-1]). No identifiable diffraction lines for Ru species could be detected, indicating that Ru NPs were small or/and of low contents and uniformly dispersed on the support of ZIF-67 [39, 41, 42].

**Figure 1:**
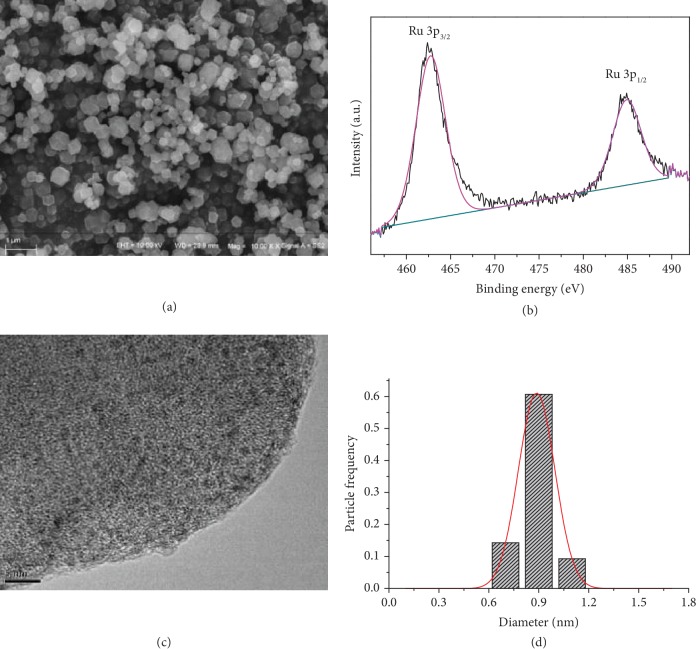
Characterization of Ru@ZIF-67 composites. (a) SEM image of catalyst *1b*; (b) XPS spectra for 3p_3/2_ and 3p_1/2_ of Ru in *1b*; (c) TEM image; (d) corresponding size distribution of Ru NPs in *1b* (0.88 ± 0.3 nm).

Scanning electron microscopy (SEM) image revealed the morphology of the obtained composite Ru@ZIF-67 as nanocubes with an average size of approx. 200 nm (Figure 1(a)), which was the same as that of ZIF-67 ([Supplementary-material supplementary-material-1]). X-ray photoelectron spectroscopy and transmission electron microscopy (TEM) images were used to further study the composition of Ru@ZIF-67. Taking *1b* as an example, XPS spectrum of *1b* (Figure 1(b) and [Supplementary-material supplementary-material-1]) showed the peaks at 462.0 eV and 485.1 eV corresponding to Ru(0) 3p_3/2_ and 3p_1/2_, respectively, while the two peaks with binding energy around 280.6 eV and 284.2 eV were attributed to 3d_5/2_ and 3d_3/2_ of Ru(0), respectively ([Supplementary-material supplementary-material-1]) [43–46]. No peaks for Ru with other valences were observed by XPS, confirming that Ru^3+^ cations were completely converted into Ru(0).

**Figure 2:**
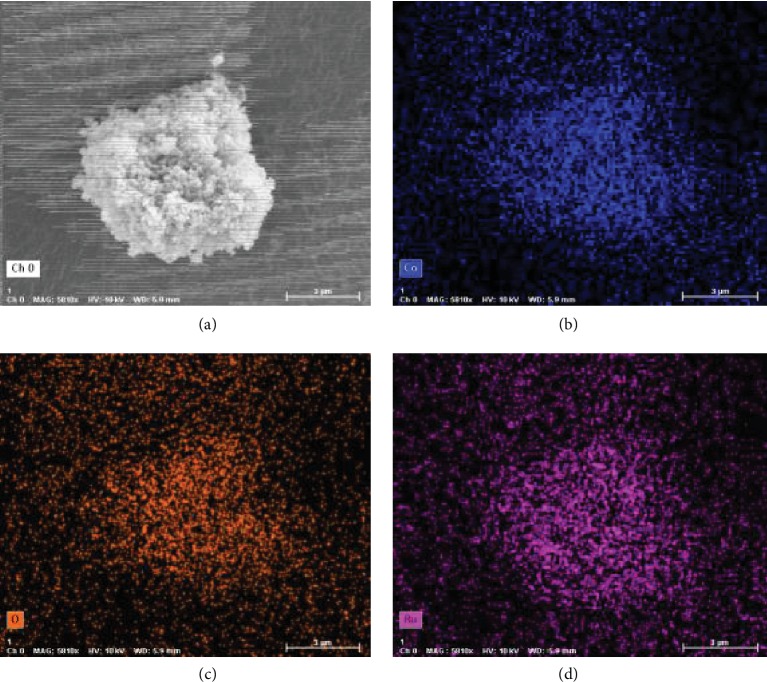
Elemental distribution maps for catalyst *1b*: (a) SEM image; (b) O; (c) Co; (d) Ru.

As shown in the TEM micrograph of *1b* (Figure 1(c)), Ru NPs were homogeneously dispersed without significant agglomeration with a uniform particle size around 0.90 nm (Figure 1(d)), which matched the internal diameter (1.14 nm) of ZIF-67's pores very well [47, 48]. This is a clear indication that the Ru NPs in *1b* located mainly inside the framework of ZIF-67, because the confinement effect of ZIF-67 limited the growth and aggregation of Ru NPs, which is accordant with the N_2_ adsorption isotherm measurement ([Supplementary-material supplementary-material-1]). Energy-dispersive spectroscopy (EDS) analyses further illustrated the uniformly distribution of Ru NPs in the pores of ZIF-67 for *1a*–*1c* (Figure 2 and Figures [Supplementary-material supplementary-material-1]).

### 2.5. Complete Hydrogenation of Xylose to Xylitol

Catalytic activities of the prepared Ru@ZIF-67 catalysts (*1a*–*1c*) were applied in the liquid phase hydrogenation of xylose to xylitol at atmospheric pressure, and various reaction conditions were screened to pursue optimized catalytic activity and selectivity (Table 1). As shown by entries 1-3, with the increase of Ru NPs loading from *1a* to *1b*, the selectivity of xylitol increased from 88.6% to 93.6%, because the increased Ru NPs could supply more active sites accessible to the substrates [11–16, 49]. However, although with higher Ru loading than *1b*, *1c* showed decreased catalytic activity with the xylitol selectivity of 91.5%, which could be attributed to the aggregation of Ru NPs in *1c* ([Supplementary-material supplementary-material-1]) due to the over increase of Ru loadings [11–16, 21–24, 49]. Subsequently, we focused on *1b* for the further survey of other catalytic parameters. When reducing the amount of *1b*, the selectivity of xylitol decreased correspondingly (entries 4 and 5) due to the less active sites at lower catalyst amount [7]. Xylose was well soluble in water, whose solubility in alcohols was quite limited [50–52], while the solubility of H_2_ in water was very low [51]. Consequently, we used a mixture of methanol and water as solvent to get the optimized catalytic performance. The different volume ratios of methanol and water were screened (entries 2, 6, and 7) to obtain the best ratio of MeOH/H_2_O as 1 : 1 (entry 6). When reducing either the reaction time or temperature, the selectivity of xylitol decreased (entries 8-10). Blank reaction illustrated that ZIF-67 itself gave no catalytic performance to produce xylitol (entry 11). Based on these surveys, we obtained the optimized condition for *1b* under 1 atm H_2_, with MeOH/H_2_O of 1 : 1 as solvent, a temperature of 50°C, and a reaction time of 48 hours. Impressively, under the optimized catalysis condition with Ru@ZIF-67 composite *1b*, no product other than xylitol could be detected, and we obtained a complete conversion of xylose to xylitol with 100% yield and selectivity.

**Figure 3:**
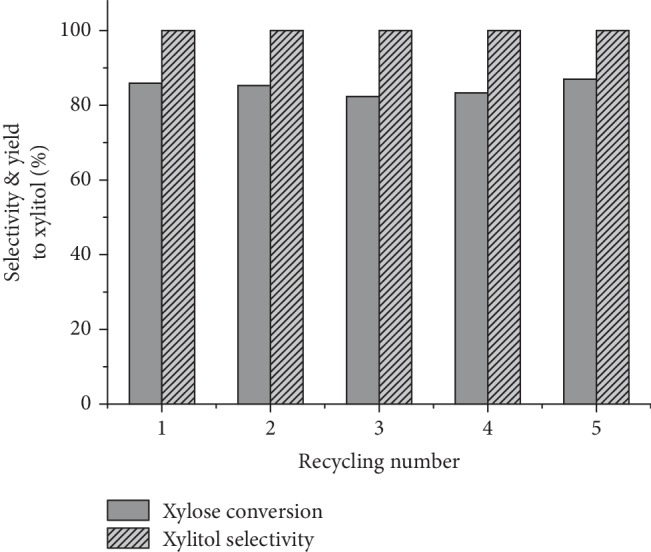
Recycling tests with the catalyst *1b* for the catalytic hydrogenation of xylose. Reaction conditions: xylose (225 mg), H_2_ (1.0 atm), methanol/water (1 : 1) (5 mL), 50°C, and 48 h.

As a heterogeneous catalyst, the durability and reusability of *1b* were tested. After being separated from the reaction mixture by filtration, *1b* was reused in successive reactions under the optimized conditions. To ensure the recycling experiments being carried out at relatively low conversion, the substrate amount was increased to 225 mg. Afterwards, the separated catalyst was reused in successive reactions under the optimized conditions. As shown in Figure 3, the conversion rate was reduced to approx. 86% due to the increase of the substrate amount from 150 to 225 mg, which was kept constant during the whole cycling tests, indicating no significant loss of catalytic activity after five successive tests. In the whole recycling process, still no other product other than xylitol could be detected, indicating the persistence of the 100% selectivity, which significantly simplified the product-purification process confirming the excellent reusability of the Ru@ZIF-67 catalyst.

Negligible loss (0.06 wt%) of Ru NPs in catalyst *1b* was observed after catalytic circles by ICP-OES analysis, demonstrating that there was no leakage of Ru NPs from the MOF pores. PXRD measurements showed the pattern of *1b* after the fifth run was still in good agreement with that of pure ZIF-67 ([Supplementary-material supplementary-material-1]), implying that the framework of ZIF-67 maintained well after the whole cyclic runs. The regenerated *1b* after the fifth run was characterized by TEM as well ([Supplementary-material supplementary-material-1]). Barely any increase in the average size of Ru NPs was observed (0.88 ± 0.3 vs. 0.90 ± 0.3 nm), indicating the maintenance of the excellent dispersity of NPs by the MOF cages (Figure 1 and [Supplementary-material supplementary-material-1]). These results further confirmed the excellent reusability of Ru@ZIF-67 during the hydrogenation reaction.

To the best of our knowledge, this is the first report on H_2_ hydrogenation of xylose under ambient pressure. To further illustrate the activity of our Ru@ZIF-67 catalyst, we summarized a comparison of our Ru@ZIF-67 composite with other supported Ru catalysts ever reported for the hydrogenation of xylose [6, 7, 9]. As shown in Table 2, relatively high energy consumption with both high temperature (120-190°C) and high pressure (5.5-16.0 MPa) is unavoidable for the other supported Ru catalysts. However, under the catalysis of Ru@ZIF-67 (*1b*), xylose hydrogenation was carried out under extremely mild conditions (0.1 MPa H_2_, 50°C) with superior conversion rate, selectivity, yield, and reusability, which should significantly reduce both economical and energic cost. Moreover, in order to make the comparison more convincing, we prepared those supported Ru catalysts according to the literatures ([Supplementary-material supplementary-material-1]) and tested their activity under the same condition for catalyst *1b* (1 atm H_2_, 50°C). However, the conversion of xylose and xylitol selectivity for these known supported Ru catalysts were remarkably inferior to that of Ru@ZIF-67, as listed in [Supplementary-material supplementary-material-1]. The results indicated the significance of MOF ZIF-67 as the support for this reaction. It is worthy to note that our Ru@ZIF-67 catalyst shows the first achievement of 100% selectivity in the hydrogenation of xylose to xylitol among all known applicable catalysts [53].

**Scheme 1:**
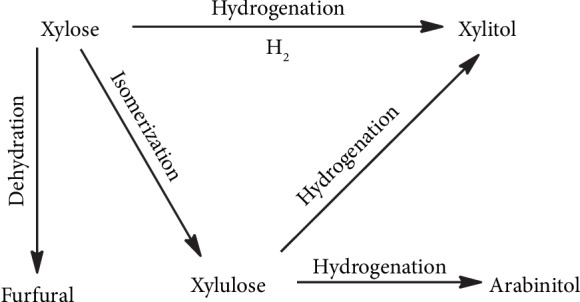
Main and side reactions in the hydrogenation of xylose.

The unexceptionable catalytic performance of Ru@ZIF-67 unambiguously originates from the combination of ZIF-67 and Ru NPs. Firstly, ZIF-67 acts as the template to homogeneously disperse Ru NPs in a uniformly ultra-small size of approx. 0.90 nm with highly improved activity, due to the long-range ordered porous structure and the confinement effect of ZIF-67. Secondly, ZIF-67 loads Ru NPs as a supporter to form the heterogeneous composite, and the pores' cage architecture of the framework separates well the Ru NPs and prevents their agglomeration during the catalytic process, which enables the thorough recirculation of Ru@ZIF-67. Thirdly, the structural flexibility of ZIF-67 makes it possible for larger substrate molecules to easily pass through the aperture [47]; therefore, the catalyst has large enough cavities to hold the molecular diameter of xylose (approx. 6.8 Å) [54]. As a “microreactor,” the framework of the catalyst provides a favorable microenvironment for the reaction of xylose and H_2_. Besides, as a MOF, the ZIF-67 matrix can capture H_2_ proactively to maintain the concentration of H_2_ around Ru NPs inside the pores, which is significantly superior to normal Ru NPs. Benefiting from the ultra-small size of Ru NPs, the high concentration of H_2_ around Ru NPs, and the continuous concentrated H_2_ molecules inside the pores of Ru@ZIF-67, we actualize the hydrogenation of xylose to xylitol under 1 atm H_2_ for the first time. As a benefit from the extreme mild reaction condition, by-products such as xylulose, arabinitol, and furfural (Scheme 1) [6, 7], whose generation is favorable at high temperature [6, 18], are significantly inhibited, and excellent selectivity and yield of xylitol are thus achieved.

### 2.6. Catalytic Hydrogenation of Acetophenone to 1-Phenylethanol

**Scheme 2:**
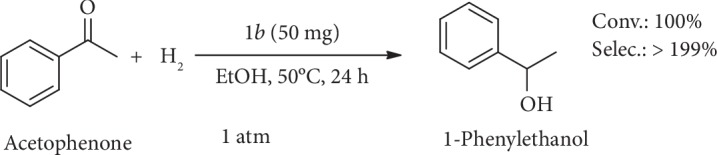
Catalytic hydrogenation of acetophenone to 1-phenylethanol by catalyst *1b*.

To further confirm the activity and universality of the Ru@ZIF-67 composite towards H_2_ hydrogenation, we examined the effect of *1b* for the hydrogenation of acetophenone to 1-phenylethanol (Scheme 2), which is also an important industrial process yet can only be manifested under high pressure till now [55–58]. Under 1 atm H_2_, *1b* exhibited excellent catalytic activity for the hydrogenation of acetophenone with 100% conversion rate and >99% selectivity of 1-phenylethanol at 50°C ([Supplementary-material supplementary-material-1]). To the best of our knowledge, this is also the first report of hydrogenating acetophenone to 1-phenylethanol atmospheric H_2_ pressure ([Supplementary-material supplementary-material-1]), which confirms the Ru@ZIF-67 composite as a highly efficient catalyst for hydrogenation and reducing the required H_2_ pressure.

## 3. Discussion

In this work, we report the perfect hydrogenation of xylose with excellent conversion rate, yield, and 100% selectivity under extreme mild reaction conditions (50°C and 1 atm of H_2_). Ultrafine-sized Ru NPs have been uniformly dispersed on ZIF-67 to afford the Ru@MOF composite, which exhibits ideal catalytic activity and durability. This is the first successful attempt for the production of one of the most popular value-added chemicals, xylitol, with 1 atm H_2_, as well as the first achievement of 100% selectivity in the hydrogenation of xylose to xylitol. The Ru@MOF composite is also proven to be ideal for the other important hydrogenation process such as the production of 1-phenylethanol from acetophenone under 1 atm H_2_ for the first time. Our result should contribute to significantly improve the industrial production of xylitol toward food, cosmetic, pharmaceutical, and synthetic resin; reduce both economy and energy costs; and lower the potential safety hazard.

## 4. Materials and Methods

### 4.1. Materials

Unless otherwise indicated, all the reagents were used without further purification: Co(NO_3_)_2_·6H_2_O (Heowns, 98%), 2-methylimidazole (Aladdin, 98%), ruthenium chloride (RuCl_3_, Meryer, 98%), xylitol (Aladdin, analytical standard), D-(+)-xylose (Aladdin, 98%), and sodium tetrahydroborate (NaBH_4_, Aldrich, 99%).

### 4.2. Characterization

The Ru loading of the as-prepared catalysts was analyzed by an inductively coupled plasma (ICP) spectrometer (PPE 8300). The thermogravimetric analysis (TGA) was conducted in N_2_ atmosphere by a Netzsch TG 209 Thermal Gravimetric Analyzer with a heating rate of 10 K/min. Powder X-ray diffraction (PXRD) was measured by the Ultima IV X-ray diffractometer using Cu K*α* radiation in the 2*θ* range of 3-50°C. The EDS was measured by a SU3500 scanning electron microscope (SEM). The morphology and composition of the catalysts were observed by a FEI Tecnai G2 F20 electron microscope (TEM). The Brunauer-Emmett-Teller (BET) surface area measurements were performed with nitrogen adsorption and desorption isotherms at 77 K using a BeiShiDe 3H-2000PM2 instrument. The isotherm of hydrogen was measured at 298 K using an Autosorb iQ Station 1 Gas Sorption Analyzer.

### 4.3. Recycling Experiments

For stability tests, Ru@MOF catalyst was recovered from the reaction mixture by centrifugation and washed with water and methanol for several times. Then, it was dried at 100°C under vacuum before reuse. To ensure the recycling experiments being carried out at relatively low conversion, the substrate amount was increased to 225 mg. Afterwards, the separated catalyst was reused in successive reactions under the optimized conditions.

### 4.4. Catalytic Mechanism

In view of the plausible mechanisms reported in the literature [6, 9], reactants (xylose and H_2_) were firstly dissolved in the solvent and then diffused to the surface of Ru@ZIF-67. Hydrogen was firstly adsorbed on the active site (Ru) and decomposed to produce active hydrogen atoms. Subsequently, the active hydrogen atom interacted with the aldehyde group of xylose molecules to form xylitol.

## Figures and Tables

**Table 1:** Hydrogenation of xylose to xylitol by Ru@ZIF-67 composites^a^.

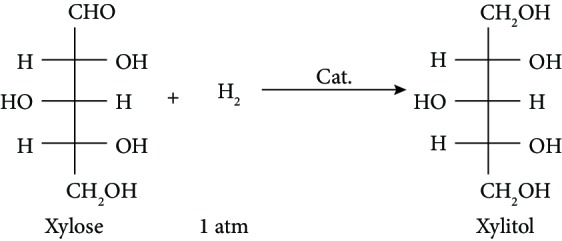						
Entry	Catalyst (mg)	Ru (mmol)^b^	Solvent	%conv.	%sel.	
				Xylose	Xylitol	Furfural
1	*1a* (100)	0.092	Methanol/water (2 : 1)	100	88.6	11.4
2	*1b* (100)	0.118	Methanol/water (2 : 1)	100	93.6	6.40
3	*1c* (100)	0.155	Methanol/water (2 : 1)	100	91.5	8.50
4	*1b* (70)	0.083	Methanol/water (2 : 1)	100	71.5	28.5
5	*1b* (50)	0.059	Methanol/water (2 : 1)	100	60.8	39.2
6	*1b* (100)	0.118	Methanol/water (1 : 1)	100	100	0
7	*1b* (100)	0.118	Methanol	100	88.3	11.7
8^c^	*1b* (100)	0.118	Methanol/water (1 : 1)	92.2	89.9	10.1
9^d^	*1b* (100)	0.118	Methanol/water (1 : 1)	100	93.0	7.00
10^e^	*1b* (100)	0.118	Methanol/water (1 : 1)	70	65.7	34.3
11	ZIF-67 (100)	0	Methanol/water (1 : 1)	15.4	0	100

^a^Reaction conditions: xylose (150 mg), H_2_ (1.0 atm), solvent (5 mL), 50°C, and 48 h. ^b^Analytical results of ICP. ^c^24 h. ^d^30 h. ^e^25°C.

**Table 2:** Comparison of catalysts for the hydrogenation of xylose to xylitol.

Catalyst	T (°C)	P(H_2_) (MPa)	Conv. (%)	Sel. (%)	Yield (%)	Ref.
Ru@ZIF-67	50	0.1	100	100	100	This work
Ru-HYZ	120	5.5	62	98	—	[7]
Ru/NiO-TiO_2_	120	5.5	99.9	99.8	99.7	[6]
Ru/C	120	5.5	96.5	97.5	94.0	[6]
Ru/TiO_2_	120	5.5	97.1	99.0	96.1	[6]
Ru/Al_2_O_3_	190	16	94	—	25	[9]

## Data Availability

All data needed to evaluate the conclusions in the paper are included in the paper and the Supplementary Materials.

## Supplementary Materials

Supplementary Materials 1. Catalyst characterization Figure S1: powder X-ray diffraction patterns of ZIF-67 and (*1a*–*1c*). Figure S2: SEM image of ZIF-67. Figure S3: XPS spectra of catalyst *1b*: (a) the survey scan, (b) Ru 3p, (c) Ru 3d, and (d) Co 2p. Figure S4: TEM image of catalyst *1a* (left) and size distribution of Ru NPs in *1a* (0.97 ± 0.3 nm) (right). Figure S5: TEM image of catalyst *1b* (left) and size distribution of Ru NPs in *1b* (0.88 ± 0.3 nm) (right). Figure S6: TEM image of catalyst *1c* (left) and size distribution of Ru NPs in *1c* (0.91 ± 0.3 nm) (right). Figure S7: TEM image of catalyst *1b* (left) after five runs and size distribution of Ru NPs in *1b* recycled (0.90 ± 0.3 nm) (right). Figure S8: N2 adsorption/desorption isotherms of ZIF-67 and *1a*–*1c* at 77 K (left). Table S1: characterization results of ZIF-67 and Ru@ZIF-67. Figure S9: elemental distribution maps for catalyst *1a*: (a) SEM image, (b) Co, (c) O, and (d) Ru. Figure S10: elemental distribution maps for catalyst *1c*: (a) SEM image, (b) Co, (c) O, and (d) Ru. Figure S11: TGA curves of ZIF-67 as-prepared and *1a*–*1c*. Table S2: catalysts for the hydrogenation of xylose to xylitol under 1 atm of H_2_. Table S3: hydrogenation of acetophenone to 1-phenylethanol by *1b*. Table S4: catalysts for the hydrogenation of acetophenone to 1-phenylethanol.
