# Supplementary material for: Toward Green Production of Chewing Gum and Diet: Complete Hydrogenation of Xylose to Xylitol over Ruthenium Composite Catalysts under Mild Conditions
Source: Research (Wash D C). 2019 Nov 29;2019:5178573. doi: 10.34133/2019/5178573 (PMC6944490; doi:10.34133/2019/5178573)
Supplement: Supplementary Materials — 1. Catalyst characterization Figure S1: powder X-ray diffraction patterns of ZIF-67 and (1a–1c). Figure S2: SEM image of ZIF-67. Figure S3: XPS spectra of catalyst 1b: (a) the survey scan, (b) Ru 3p, (c) Ru 3d, and (d) Co 2p. Figure S4: TEM image of catalyst 1a (left) and size distribution of Ru NPs in 1a (0.97 ± 0.3 nm) (right). Figure S5: TEM image of catalyst 1b (left) and size distribution of Ru NPs in 1b (0.88 ± 0.3 nm) (right). Figure S6: TEM image of catalyst 1c (left) and size distribution of Ru NPs in 1c (0.91 ± 0.3 nm) (right). Figure S7: TEM image of catalyst 1b (left) after five runs and size distribution of Ru NPs in 1b recycled (0.90 ± 0.3 nm) (right). Figure S8: N2 adsorption/desorption isotherms of ZIF-67 and 1a–1c at 77 K (left). Table S1: characterization results of ZIF-67 and Ru@ZIF-67. Figure S9: elemental distribution maps for catalyst 1a: (a) SEM image, (b) Co, (c) O, and (d) Ru. Figure S10: elemental distribution maps for catalyst 1c: (a) SEM image, (b) Co, (c) O, and (d) Ru. Figure S11: TGA curves of ZIF-67 as-prepared and 1a–1c. Table S2: catalysts for the hydrogenation of xylose to xylitol under 1 atm of H2. Table S3: hydrogenation of acetophenone to 1-phenylethanol by 1b. Table S4: catalysts for the hydrogenation of acetophenone to 1-phenylethanol. [file 5178573.f1.docx]

Supplementary Materials

1. Catalyst Characterization

Figure S1. Powder X-ray diffraction patterns of ZIF-67 and (**1a**-**1c**).


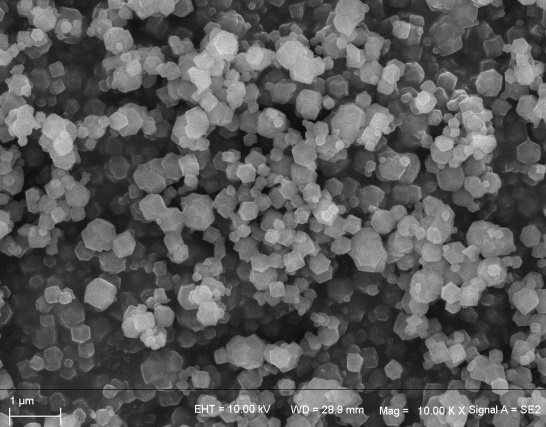


Figure S2. SEM image of ZIF-67.

Figure S3. XPS spectra of catalyst **1b**: (a) the survey scan, (b) Ru 3p, (c) Ru 3d, (d) Co 2p.

The X-ray photoelectron spectroscopy (XPS) supplied further insights to the metal electronic states of catalyst **1b**. As shown in Figure S3a, the main peaks were assigned to C 1s, N 1s, Ru 3p, Ru 3d, O 1s and Co 2p, respectively. Figure S3b showed the peaks observed at 462.0 and 485.1eV were in good agreement with the values of Ru(0) 3p_3/2_ and 3p_1/2_, separately. Figure S3c showed two peaks with binding energy around 280.6eV and 284.2eV, which were attributed to the 3d_5/2_ and 3d_3/2_ of Ru(0), respectively [43-46]. Moreover, Figure S3d showed two peaks at 779.5 eV and 794.6 eV which referred to the Co^3+^ species in ZIF-67, whereas the peaks at 783.2 eV and 798.6 eV were assigned to Co^2+^ species in ZIF-67 [1]. The above results confirmed that all of the Ru^3+^ cations were completely converted into Ru(0).

Figure S4. TEM image of catalyst **1a** (left) and size distribution of Ru NPs in **1a** (0.97 ± 0.3 nm) (right).

Figure S5. TEM image of catalyst **1b** (left) and size distribution of Ru NPs in **1b** (0.88 ± 0.3 nm) (right).

Figure S6. TEM image of catalyst **1c** (left) and size distribution of Ru NPs in **1c** (0.91 ± 0.3 nm) (right).

Figure S7. TEM image of catalyst **1b** (left) after five runs and size distribution of Ru NPs in **1b** recycled (0.90 ± 0.3 nm) (right).

Figure S8. N_2_ adsorption/desorption isotherms of ZIF-67 and **1a**-**1c** at 77 K (left).

N_2_ adsorption-desorption isotherms of ZIF-67 and **1a**-**1c** at 77 K were showed in Figure S8 and the characterization results of catalysts were summarized in Table S1. Compared with ZIF-67, the BET surface areas and pore volumes of catalysts Ru@ZIF-67 were remarkably reduced, which should be due to the fact that the pores of ZIF-67 might be occupied by dispersed Ru NPs and/or blocked by the Ru NPs deposited at framework surface of ZIF-67. However, catalyst **1c** with higher Ru loading showed a slight increase in surface area, which may be ascribed to a lower occupation of the cavities by the aggregated Ru NPs [2].

Table S1. Characterization results of ZIF-67 and Ru@ZIF-67.

| Entry | Catalyst | S_BET_ (m^2^·g^-1^) | Langmuir (m^2^·g^-1^) | *V*_tot_ (cm^3^·g^-1^) | Ru (wt %) |
| --- | --- | --- | --- | --- | --- |
| 1 | ZIF-67 | 1755 | 1913 | 1.250 | 0 |
| 2 | **1a** | 1350 | 1537 | 0.781 | 9.30 |
| 3 | **1b** | 1145 | 1268 | 0.744 | 11.9 |
| 4 | **1c** | 1567 | 1693 | 1.199 | 15.6 |

Figure S9. Elemental distribution maps for catalyst **1a**: (a) SEM image, (b) Co, (c) O, (d) Ru.

Figure S10. Elemental distribution maps for catalyst **1c**: (a) SEM image, (b) Co, (c) O, (d) Ru

Figure S11. TGA curves of ZIF-67 as-prepared and (**1a**-**1c**).

Table S2. Catalysts for the hydrogenation of xylose to xylitol under 1 atm of H_2_.^a^

| Catalyst | Ru (mmol)^b^ | Conv.(%) | Sel.(%) | Ref. |
| --- | --- | --- | --- | --- |
| Ru@ZIF-67 | 0.118 | 100 | 100 | this work |
| Ru-HYZ | 0.107 | 51.0 | 50.9 | [3] |
| Ru/C | 0.093 | 0 | 0 | [4] |
| Ru/TiO_2_ | 0.113 | 1.29 | — | [4] |

^a^Reaction conditions: xylose (150 mg), H_2_ (1.0 atm), solvent (5 mL), 50 °C, 48 h.

^b^Analytical results of ICP.

Table S3. Hydrogenation of acetophenone to 1-phenylethanol by **1b**.

| Catalyst | Time (h) | Temperature (°C) | Conv. (%) | Sele.(%) |
| --- | --- | --- | --- | --- |
| **1b** | 12 | 60 | 100 | 97.53 |
| **1b** | 12 | 30 | 100 | 82.4 |
| **1b** | 24 | 60 | 100 | 98.12 |
| **1b** | 24 | 50 | 100 | 99.5 |
| ZIF-67 | 12 | 30 | 0 | — |

Reaction conditions: acetophenone (0.4 mmol), H_2_ (1.0 atm), catalyst (50 mg)

and EtOH (3 mL).

Table S4. Catalysts for the hydrogenation of acetophenone to 1-phenylethanol.

| Catalyst | T (°C) | P(H_2_) | Conv. (%) | Selc. (%) | Ref. |  |
| --- | --- | --- | --- | --- | --- | --- |
| Ru@ZIF-67 | 50 | 0.1 MPa | 100 | >99 | this work |  |
| Pd/PPh_3_@FDU-12 | 60 | 4 bar | >99 | >99 | [5] |  |
| Pd/PSiO_2_ | 60 | 20 bar | 100 | >99.9 | [6] |  |
| Pd NPs | 20 | 1.5 bar | 100 | 90.1 | [7] | |
| Ru-TPP-(1R,2R)-DPEN | 25 | 2.0 MPa | 99.9 | 75.1 | [8] |  |

References

[1] D. D. Tuan, K.-Y. A. Lin, “Ruthenium supported on ZIF-67 as an enhanced catalyst for hydrogen generation from hydrolysis of sodium borohydride,” *Chemical Engineering Journal*, vol. 351, pp. 48-55, 2018.

[2] X.-H. Liu, J.-G. Ma, Z. Niu, G.-M. Yang, P. Cheng, “An efficient nanoscale heterogeneous catalyst for the capture and conversion of carbon dioxide at ambient pressure," *Angewandte Chemie International Edition*, vol. 54, no. 3, pp. 988-991, 2015.

[3] D. K. Mishra, A. A. Dabbawala, J. S. Hwang, “Ruthenium nanoparticles supported on zeolite Y as an efficient catalyst for selective hydrogenation of xylose to xylitol,” *Journal of Molecular Catalysis A: Chemical*, vol. 376, pp. 63-70, 2013.

[4] M. Yadav, D. K. Mishra, J. S. Hwang, “Catalytic hydrogenation of xylose to xylitol using ruthenium catalyst on NiO modified TiO_2_ support,” *Applied Catalysis A: General*, vol. 425-426, pp. 110-116, 2012.

[5] M. Guo, H. Li, Y. Ren, X. Ren, Q. Yang, C. Li, “Improving catalytic hydrogenation performance of Pd nanoparticles by electronic modulation using phosphine ligands,” *ACS Catalysis*, vol. 8, no. 7, pp. 6476-6485, 2018.

[6] S. Jayakumar, A. Modak, M. Guo, H. Li, X. Hu, Q. Yang, “Ultrasmall Platinum stabilized on triphenylphosphine-modified silica for chemoselective hydrogenation,” *Chemistry A European Journal*, vol. 23, no. 32, pp. 7791-7797, 2017.

[7] Y. Yuan, Y. V. Kaneti, X. Jiang, J. Huang, A. Yu, “Seed-mediated synthesis of dendritic platinum nanostructures with high catalytic activity for aqueous-phase hydrogenation of acetophenone,” *Journal of Energy Chemistry*, vol. 24, no. 5, pp. 660-668, 2015.

[8] L. Ye, H. Lin, H. Zhou, Y. Yuan, “Support and size effects of ruthenium catalysts with a chiral modifier for asymmetric hydrogenation of aromatic ketones,” *The Journal of Physical Chemistry C*, vol. 114, no. 46, pp. 19752-19760, 2010.
